# Irony Understanding in Children With Attention-Deficit/Hyperactivity Disorder: A Comparative Analysis

**DOI:** 10.7759/cureus.53892

**Published:** 2024-02-09

**Authors:** Regina Pinto Silva, Bárbara Mota, Linda Candeias, Victor Viana, Micaela Guardiano

**Affiliations:** 1 Pediatrics, Centro Hospitalar Universitário de São João, Porto, PRT; 2 Psychology, Prisma – Therapies and Development Center, Porto, PRT; 3 Psychology, Centro Hospitalar Universitário de São João, Porto, PRT

**Keywords:** neurodevelopment, executive functions, children, adhd, irony

## Abstract

Introduction: Attention-deficit/hyperactivity disorder (ADHD) is a neurodevelopmental disorder characterized by persistent patterns of inattention, hyperactivity, and impulsivity that can significantly impact a child's daily life and academic performance. Some studies have noted challenges in social cognition among children with ADHD, specifically in aspects like emotion perception and processing, empathy, Theory of Mind, and pragmatics. One of the lesser-explored aspects of ADHD is its potential impact on higher-level language skills, such as irony understanding. Our goal in this study was to compare irony comprehension in two groups: schoolchildren with normal development and schoolchildren with ADHD.

Methods: We performed a comparative cross-sectional study on a total of 35 children aged between 6 and 12 years: 17 with ADHD and 18 not neurodevelopmentally impaired. Irony comprehension was evaluated using an assessment method validated for the Portuguese population: Turtle on the Island - Battery of Assessment of Executive Functions in Children (TI-BAFEC). We also applied the EACE (Emotional Awareness and Coping in Children) scale. It is the Portuguese adaptation of the Assessment of Children's Emotion Skills and assesses children's emotional knowledge.

Results: We obtained two comparable groups concerning sociodemographic data. The analysis of the data, using the Mann-Whitney U Test, showed that in all parameters, the control group consistently exhibited superior performance compared to the ADHD group. However, only TI-BAFEC (p = 0.005) and emotional behavior (p = 0.007) showed a significant statistical variance between the two groups.

Conclusion: This article shows that children with ADHD encounter challenges in grasping irony. However, the underlying cognitive processes contributing to these difficulties warrant further investigation.

## Introduction

Attention-deficit/hyperactivity disorder (ADHD) is a neurodevelopmental disorder characterized by persistent patterns of inattention, hyperactivity, and impulsivity that can significantly impact a child's daily life and academic performance [1]. It is one of the most prevalent neurodevelopmental disorders in childhood; its prevalence in the pediatric age group is around 5-7% and is higher in males. ADHD not only impacts cognitive and academic aspects but also extends its effects into the realm of social functioning [2,3,4].

Children diagnosed with ADHD often exhibit deficient social behavior and compromised social cognition [5]. Social cognition encompasses various components such as understanding others' thoughts, perceiving emotions, empathizing, attributing false beliefs, and grasping intended meanings, among other facets [6]. Despite clinical observations indicating deficits in social cognition among individuals with ADHD, research in this domain remains limited, creating a notable gap that the present study aimed to address. Certain studies have noted challenges in social cognition among children with ADHD, specifically in aspects like emotion perception and processing, empathy, Theory of Mind (ToM), and pragmatics [7-9].

One of the lesser-explored aspects of ADHD is its potential impact on higher-level language skills, such as irony understanding. Irony is a complex form of figurative language that involves conveying a meaning that is opposite to the literal interpretation of the words used [10].

The underlying mechanisms of both spoken and written expressions of irony remain a subject of controversy. Six distinct processing theories have emerged: the standard pragmatic view (SPV), echoic mention theory (EMT), pretense theory (PT), allusion pretense theory (APT), the echoing-contrasting cognitive operation (ECOP) model, and the parallel constraint-satisfaction (PCS) approach [11].

The ability to comprehend irony among typically developing individuals starts to develop around the ages of five to six years, and this skill continues to advance through the middle school years, spanning ages 13 to 16 [12].

Both Ackerman [13] and Hancock et al. (2000) [14] assert that understanding irony involves two primary processes and skills. The first among these is the capacity to recognize the indirect meaning and its contrast with the situation in which it is used. The second is the skill to deduce the metaphorical aspect of irony based on the tone of speech. These skills have been directly associated with tasks related to recognizing false beliefs and ToM. This implies that they play a significant role in successfully comprehending expressions of irony.

This article aims to explore and compare irony understanding in children with and without ADHD, shedding light on whether there exists a statistically significant difference between the two groups.

## Materials and methods

Participants and procedures

To examine the relationship between ADHD and irony understanding, we conducted a comparative cross-sectional study involving a sample of 35 children: 17 children with ADHD and 18 children with normal neurodevelopment. Their ages varied from 6 to 12 years. This study was approved by the Ethics Committee of Centro Hospitalar Universitário de São João (CHUSJ). Informed parental consent was obtained for all children.

Children in the ADHD group were recruited from the medical records of the Pediatrics Neurodevelopment Unit of CHUSJ. The inclusion criteria were pediatric patients diagnosed with ADHD, without associated comorbidities, aged between 6 and 12 years, attending the neurodevelopmental consultation at HSJ (Hospital São João), and those who agreed to enter the study. Their diagnosis had been made by a team of qualified clinicians using the Diagnostic and Statistical Manual of Mental Disorders, Fifth Edition (DSM-5) criteria. Of the 17 children with ADHD, one was not receiving medication, and 16 were treated with methylphenidate. Parents were instructed to discontinue medication on the day of evaluation, allowing a 24-hour period to elapse since the last administered dose. We excluded from our study children who had a diagnosis of psychiatric or neurodevelopmental disorder, cognitive impairment, or motor/sensory deficits.

Children in the control group (typically developing group) were recruited through primary care. The inclusion criteria for these groups were children aged between 6 and 12 years, who had never met the criteria for ADHD, and those who agreed to enter the study. We excluded those who have a diagnosis of other psychiatric or neurodevelopmental disorders, cognitive impairment, or motor/sensory deficits.

The administered tests were conducted under the oversight of the same medical professional (psychologist) and with parental presence, lasting an average of 20 to 30 minutes per evaluation. In terms of sociodemographic information, we gathered data pertaining to gender, age, and mother's educational background.

Materials

The evaluation of irony understanding was conducted using a validated assessment approach for 6- to 13-year-old children, designed for the Portuguese population: "Turtle on the Island - Battery of Assessment of Executive Functions in Children" (Tartaruga na ilha - Bateria de Avaliação de Funções Executivas em Crianças (TI-BAFEC)) [15]. This method was developed to complement the assessment of Executive Functions in the Portuguese population, with a specific focus on the pediatric context, where there was a lack of instruments available in European Portuguese for this purpose. It is an instrument specifically designed to assess executive functions in children, validated for the Portuguese population, that is completed within 25 minutes. The TI-BAFEC aims to highlight the most recent aspects of research in the field of Executive Functions, integrating both the cognitive and emotional aspects of executive functions in its assessment objectives. The instrument is presented to the child in the form of a story containing games, resembling a small book. It consists of 14 tests organized into three domains: Cognitive Executive Functions, which include tests assessing verbal fluency, attention, memory, and planning; Emotional Executive Functions, which include tests evaluating ToM, understanding irony, and emotional decision-making; and finally, the Interaction between Cognitive and Emotional Executive Functions, which involves direction and flexibility, that is, the ability to change strategies. Each test provides an independent score, allowing for comparison with age-specific percentiles.

The irony understanding evaluation method consisted of the ability of the children to access the nonliteral meaning of language (evaluated through humorous stories). A situation was presented in which one of the characters made an ironic comment. After the presentation of the story, the child was questioned about reality (focusing on understanding the situation and the comment) and about what the character who made the comment actually thought of the situation. With the score obtained, we were able to objectify the children's ability to understand irony.

We also applied the Emotional Awareness and Coping in Children (EACE) scale. It is the Portuguese adaptation of the Assessment of Children's Emotion Skills and assesses children's emotional knowledge. It consists of three subscales: Facial Expressions (20 items), Emotional Situations (15 items), and Emotional Behaviors (15 items). The aim is to evaluate the child's ability to correctly associate one of five possible feelings, that is, joy, sadness, fear, anger, or neutral ("no feeling"), with each stimulus. It is administered by presenting a series of images depicting emotional expressions, emotional/social situations, and emotional/social behaviors to the child. The goal is to evaluate the child's ability to correctly associate one of five possible feelings, happy, sad, scared, angry, or neutral ("no feeling") with each stimulus [16,17].

Statistical analysis

Statistical analysis was conducted using IBM SPSS Statistics for Windows, Version 27 (Released 2020; IBM Corp., Armonk, New York). The significance level was set at p < 0.05. Statistical analysis was performed using non-parametric tests, namely, the Mann-Whitney U Test. Descriptive and comparative analyses were performed on the collected sociodemographic data using the t-test. When analyzing the results, the mean values and standard deviation values were considered.

## Results

In Table 1, we can see that this study included 17 patients in the ADHD group and 18 in the control group. In the ADHD group, 12 (70.6%) were male and 5 (29.4%) were female, while in the control group, 8 (44.4%) were male and 10 (55.6%) were female. Regarding the mother's educational level, 3 (17.6%) completed the fifth/sixth grade, 4 (23.5%) completed the seventh/ninth grade, 4 (23.5%) completed high school, and 6 (35.3%) attended university. This is in comparison to 3 (16.7%), 6 (33.3%), 3 (16.7%), and 6 (33.3%), respectively, in the control group.

Examining Table 1, we observe that the average age in the ADHD group is 8.9 years, while in the control group, it is 8.4 years, signifying the absence of a statistically significant difference between the two groups. Similarly, in terms of sex distribution and maternal education, no statistically significant differences were identified. Therefore, concerning sociodemographic data, the two groups can be considered comparable.

**Table 1:** Analysis of sociodemographic data of children and mothers Statistical analysis using the t-test ADHD: attention-deficit/hyperactivity disorder

	N (%)		p-value
	ADHD	Control	
Sex			0.125
Female	5 (29.4)	10 (55.6)	
Male	12 (70.6)	8 (44.4)	
Age (mean)	8.9	8.4	0.317
Mother’s education			0.801
Primary school	0	0	
5–6th grade	3 (17.6)	3 (16.7)	
7–9th grade	4 (23.5)	6 (33.3)	
High school	4 (23.5)	3 (16.7)	
University	6 (35.4)	6 (33.3)	

In Table 2, we present the average results in the control group and the ADHD group, along with the corresponding standard deviations in the results obtained in the following tests: TI-BAFEC - irony domain, facial expressions, emotional behavior, and complete emotional perceptions. We also provide the value of the statistical analysis result using the Mann-Whitney U test.

As seen in Table 2, analysis of the data, using the Mann-Whitney U Test, illuminated that in all parameters the control group consistently exhibited superior performance compared to the ADHD group. However, only the TI-BAFEC - irony domain and emotional behavior showed a significant statistical variance between the two groups.

**Table 2:** Comparison of TI-BAFEC - irony domain result, behavior, comprehension of facial expressions, and complete emotional perception in the different groups SD: standard deviation, U: Mann-Whitney U-test, ADHD: attention-deficit/hyperactivity disorder, TI-BAFEC: Turtle on the Island - Battery of Assessment of Executive Functions in Children, EACE: Emotional Awareness and Coping in Children TI-BAFEC (min-max: 0-15); EACE – Facial Expression (min-max: 0-16); EACE – Emotional Behavior (min-max: 0-12); EACE – Complete Emotional Perception (min-max: 0-38)

	Mean					
	ADHD	SD	Control	SD	U	p
TI-BAFEC (Irony Domain)	6	2.921	8.85	4.038	149	0.005
EACE – Facial Expressions	15	1.592	15.75	1.712	124	0.205
EACE – Emotional Behavior	9.50	1.095	10.69	0.947	164.5	0.007
EACE – Complete Emotional Perception	31.06	3.276	32.17	2.588	117	0.347

## Discussion

The primary aim of our study was to compare the outcomes of irony comprehension assessments between school-aged children with ADHD and those with typical neurodevelopment within the Portuguese population. To our knowledge, this is the first study of this kind in the Portuguese population.

The findings of this study raise intriguing questions about the potential cognitive mechanisms underlying the difficulty children with ADHD experience in understanding irony. One possible explanation could be related to the executive function deficits commonly associated with ADHD [18]. Executive functions, including working memory, cognitive flexibility, and inhibitory control, play a crucial role in processing complex language cues, such as those found in ironic statements [19]. Children with ADHD often struggle with these executive functions, which might hinder their ability to navigate the subtleties of ironic language effectively. It is known that understanding irony is crucial for normal development and socialization, underscoring the importance of this study [20]. It could be perceived as something of minor relevance to one's future life, but that is not the case. Appreciating irony plays a significant role in shaping interpersonal communication and fostering a nuanced understanding of language. This ability contributes to social cohesion, empathy, and a deeper comprehension of the subtleties of human interaction [21]. Therefore, exploring the nuances of irony is not just an academic pursuit; it directly impacts the way individuals navigate and engage with the complexities of the social world, highlighting its far-reaching implications for personal and social development.

Furthermore, the reduced attentional capacities of children with ADHD might contribute to their diminished ability to discern the contextual cues necessary for grasping irony. The rapid-paced nature of verbal irony demands an acute awareness of social context and the speaker's intentions [22]. Children with ADHD might find it challenging to allocate the necessary cognitive resources to fully comprehend such nuanced linguistic interactions.

It is important to note that ADHD has been connected in the literature to challenges in comprehending various forms of figurative language, including irony. This connection has been demonstrated in studies such as those by Humphries et al. [23], Bishop and Baird [24], and Bignell and Cain [25]. For instance, Caillies et al. [26] carried out a comparative investigation involving children with ADHD and their typically developing (TD) peers. Both groups underwent assessments related to ToM (specifically a second-order false belief task) and executive functions. Additionally, they listened to narratives containing instances of ironic expressions and were subsequently required to answer questions about the intentions, beliefs, and attitudes of the speakers in the stories. The outcomes of this study indicated that individuals with ADHD indeed encountered difficulties in both the ToM task and in grasping the meaning behind ironic expressions. These findings align with the conclusions drawn by Buitelaar et al. [27], who similarly proposed that a delay in ToM development impacts one's ability to comprehend irony.

Moreover, the challenges that children with ADHD encounter in grasping irony could be linked to the deficits in executive functions, as supported in a study conducted by Szamburska-Lewandowska et al. [28].

The diminished attentional capacity inherent in ADHD could also contribute to the observed discrepancies in irony comprehension. The rapid pace of verbal irony demands heightened attention to contextual cues and speaker intentions, a demand that might stretch the cognitive resources of children with ADHD.

Regarding our results, we have two comparable groups, since no significant differences were demonstrated between age, sex, and parents' education. Our analysis showed a statistically significant discrepancy in understanding between children with ADHD and their typically developing peers. While the control group consistently demonstrated a superior ability to grasp the intended ironic meaning in various scenarios, the ADHD group struggled, particularly with subtler forms of irony. These findings suggest that ADHD may indeed influence higher-level language skills, shedding light on the interconnectedness of cognitive processes, linguistic comprehension, and neurodevelopmental disorders. However, we must take into account that our study has a limitation in having a small sample size.

## Conclusions

In conclusion, this study aimed to investigate the comprehension of irony in ADHD children in comparison to children with normal neurodevelopment. The results demonstrate that children with ADHD encounter challenges in grasping irony. However, the underlying cognitive processes contributing to these difficulties warrant further investigation. Our study contributes to the existing understanding of the social cognition abilities in children with ADHD.

In the future, studies should be conducted encompassing a larger sample size. This approach would allow more comprehensive conclusions to be drawn regarding irony comprehension, explore the underlying mechanisms, and design effective interventions that promote language development and social communication skills in children with ADHD.
